# CAR-T cell therapy in multiple myeloma: Current limitations and potential strategies

**DOI:** 10.3389/fimmu.2023.1101495

**Published:** 2023-02-20

**Authors:** Xiaomin Zhang, Hui Zhang, Huixuan Lan, Jinming Wu, Yang Xiao

**Affiliations:** ^1^ Department of Hematology, Jinshazhou Hospital of Guangzhou University of Chinese Medicine, Guangzhou, China; ^2^ School of Medicine, Jishou University, Jishou, China; ^3^ School of Traditional Chinese Medicine, Southern Medical University, Guangzhou, China; ^4^ Department of Hematology, Shenzhen Qianhai Shekou Pilot Free Trade Zone Hospital, Shenzhen, China

**Keywords:** CAR-T cell therapy, antigen escape, immunosuppressive tumor microenvironment, combinatorial therapy, CAR-T cell exhaustion, relapse

## Abstract

Over the last decade, the survival outcome of patients with multiple myeloma (MM) has been substantially improved with the emergence of novel therapeutic agents, such as proteasome inhibitors, immunomodulatory drugs, anti-CD38 monoclonal antibodies, selective inhibitors of nuclear export (SINEs), and T cell redirecting bispecific antibodies. However, MM remains an incurable neoplastic plasma cell disorder, and almost all MM patients inevitably relapse due to drug resistance. Encouragingly, B cell maturation antigen (BCMA)-targeted chimeric antigen receptor T (CAR-T) cell therapy has achieved impressive success in the treatment of relapsed/refractory (R/R) MM and brought new hopes for R/R MM patients in recent years. Due to antigen escape, the poor persistence of CAR-T cells, and the complicated tumor microenvironment, a significant population of MM patients still experience relapse after anti-BCMA CAR-T cell therapy. Additionally, the high manufacturing costs and time-consuming manufacturing processes caused by the personalized manufacturing procedures also limit the broad clinical application of CAR-T cell therapy. Therefore, in this review, we discuss current limitations of CAR-T cell therapy in MM, such as the resistance to CAR-T cell therapy and the limited accessibility of CAR-T cell therapy, and summarize some optimization strategies to overcome these challenges, including optimizing CAR structure, such as utilizing dual-targeted/multi-targeted CAR-T cells and armored CAR-T cells, optimizing manufacturing processes, combing CAR-T cell therapy with existing or emerging therapeutic approaches, and performing subsequent anti-myeloma therapy after CAR-T cell therapy as salvage therapy or maintenance/consolidation therapy.

## 1 Introduction

Multiple myeloma (MM) is a plasma cell malignancy characterized by the clonal proliferation of malignant plasma cells in the bone marrow, accompanied by the excessive production of monSoclonal immunoglobulin protein (called M-protein) and subsequent end-organ damage, and it accounts for approximately 10% of hematological malignancies. With the increasing understanding of MM pathogenesis and the application of novel therapeutic agents, such as proteasome inhibitors (bortezomib, ixazomib, and carfilzomib), immunomodulatory drugs (thalidomide, lenalidomide, and pomalidomide), and monoclonal antibodies (daratumumab, isatuximab, and elotuzumab), as well as the selective inhibitors of nuclear export (selinexor), the survival outcomes of MM patients have been greatly improved (1). However, almost all MM patients eventually relapse, and especially those relapsed or refractory (R/R) patients with extramedullary disease (EMD) or high-risk cytogenetic abnormalities, such as t(4;14), t(14;16), t(14;20), gain (1q), del(17p), and TP53 mutation, as well as double/triple hit, usually have a poor prognosis. In addition, clonal evolution of MM cells under the selective pressure of treatment occurs frequently, which could result in disease progression and resistance to conventional therapy (2). Thus, novel therapeutic approaches are urgently needed for the treatment of R/R MM.

In recent years, chimeric antigen receptor T (CAR-T) cell therapy has emerged as a highly promising immunotherapy, and it has profoundly changed the treatment landscape of hematological malignancies. To generate CAR-T cells which could specifically recognize tumor surface antigens, T cells from patients or healthy donors are genetically modified with a specific tumor-targeted receptor, which is known as chimeric antigen receptor (CAR). The CAR structure contains a single chain variable fragment (scFv), which results in specific recognition of tumor surface antigens without MHC-restricted antigen presentation. Similar to effector T cells, CAR-T cells could also mediate tumor killing in several manners, including secretion of cytotoxic granules containing perforin and granzymes, production of pro-inflammatory cytokines like IFN-γ and TNF-α, and activation of Fas/Fas ligand (Fas/FasL) pathway. At present, B cell maturation antigen (BCMA) is the most successful target used for CAR-T cell therapy in MM, and anti-BCMA CAR-T cell therapy has achieved unprecedented responses in R/R MM patients and brought new hope for these R/R MM patients (3–7). In addition, R/R MM patients with EMD could also benefit from anti-BCMA CAR-T cell therapy, but these patients usually had a shorter progression-free survival (PFS) and overall survival (OS) compared with non-EMD patients (8, 9). To date, two anti-BCMA CAR-T cell products, idecabtagene vicleucel (ide-cel) and ciltacabtagene autoleucel (cilta-cel), have been approved by the US Food and Drug Administration (FDA) for the treatment of R/R MM. As an increasing number of CAR-T cell clinical trials are performed in recent years, CAR-T-related adverse events have been gradually recognized and are generally manageable, such as cytokine release syndrome, CAR-T-cell-related encephalopathy syndrome, cytopenia, and infections. In particular, due to humoral immunodeficiency of MM patients and subsequent B cell aplasia mediated by lymphodepleting chemotherapy and anti-BCMA CAR-T cell therapy, these patients are highly susceptible to infections (10, 11), especially bacterial infections (12). Therefore, immunoglobulin supplementation and prophylactic anti-infective treatment are extremely necessary for these immune-compromised patients. Nevertheless, there still remain several substantial challenges, such as the resistance to anti-BCMA CAR-T cell therapy, and the limited accessibility of CAR-T cell therapy. Thus, many research efforts are underway to explore effective strategies.

## 2 Resistance to anti-BCMA CAR-T cell therapy in multiple myeloma and potential strategies

Despite the encouraging outcomes of anti-BCMA CAR-T cell therapy in R/R MM, it usually exhibits short-term efficacy and many MM patients still experience disease recurrence or progression. The resistance mechanisms are closely related to the interactions among anti-BCMA CAR-T cells, tumor cells and the complicated tumor microenvironment, involving antigen escape and CAR-T cell exhaustion. There are several potential strategies to overcome the resistance to CAR-T cell therapy, including utilizing dual-targeted CAR-T cells and armored CAR-T cells, inhibiting intracellular exhaustion-related signals through small molecule drugs and genetic modifications, and employing bridging therapy, as well as selecting T cells collected in the early stages of disease for CAR-T cell manufacturing.

### 2.1 Overcoming antigen escape

**Figure 1:**
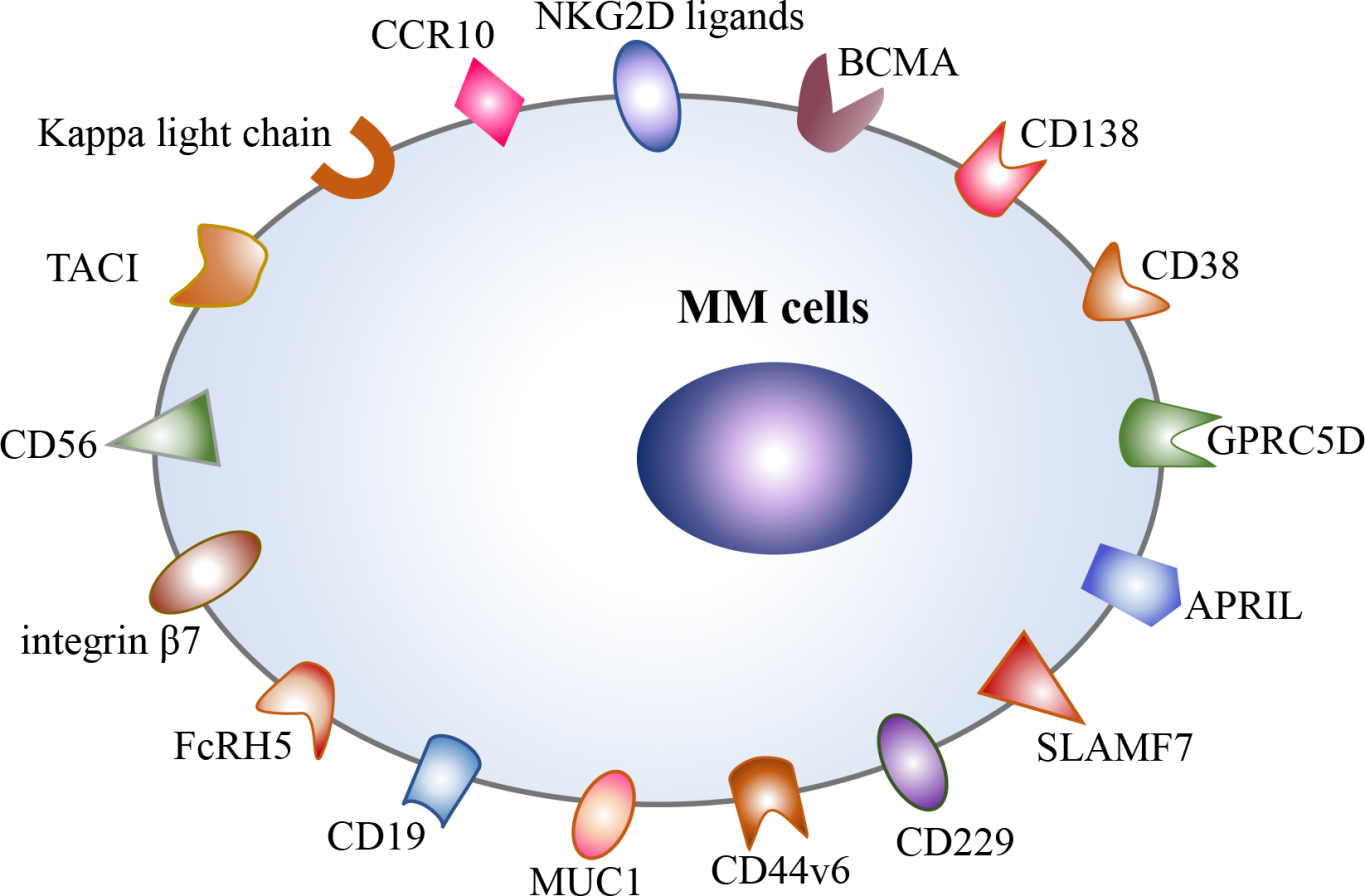
Potential therapeutic targets in multiple myeloma, including BCMA, CD138, CD38, CD19, GPRC5D, SLAMF7, APRIL, TACI, CD229, CD56, CD44v6, integrin β7, MUC1, FcRH5, Kappa light chain, CCR10, and NKG2D ligands.

Currently, BCMA is the most intensively studied target for the treatment of MM, including anti-BCMA CAR-T cell therapy and bispecific antibodies targeting BCMA and CD3, such as teclistamab (13, 14). However, a majority of MM patients still experience relapse after anti-BCMA CAR-T cell therapy (5). One of the main mechanisms is antigen downregulation or antigen loss under therapeutic pressure (15–17). Thus, targeting different surface antigens is an effective strategy to prevent antigen-negative escape, and multiple alternative targets are continuously being identified at present, including CD138, CD38, CD19, GPRC5D, SLAMF7(CS1), APRIL, TACI, CD229, CD56, MUC1, NKG2D ligands, integrin β7, Kappa light chain, FcRH5, CCR10, and CD44v6 (**Figure 1**). Most of the above targets are still in the preclinical stage (18–23), and only a few targets are explored in clinical trials, such as CD138, CD38, CD19, GPRC5D, SLAMF7, and integrin β7 (24–27) (NCT03778346). Among them, GPRC5D is the most potential target for CAR-T cell therapy in R/R MM patients at present (24, 28, 29). Recent, two phase 1 trials have reported the encouraging efficacy of anti-GPRC5D CAR-T cell therapy. In a phase 1 dose-escalation study, 17 R/R MM patients received anti-GPRC5D CAR-T cell infusion at four dose levels and 71% of them achieved a clinical responses (24). In another single-center, phase 1 trial, 10 R/R MM patients were treated with anti-GPRC5D CAR-T cells (OriCAR-017), and 100% of them showed clinical responses and 60% of them achieved a stringent complete response (sCR) (29). More importantly, these anti-GPRC5D CAR-T cells were also effective in R/R MM patients who were refractory to previous anti-BCMA CAR-T cell therapy (24, 29). However, due to the relatively short median follow‐up time, the efficacy and safety of anti-GPRC5D CAR-T cell therapy in R/R MM remain to be evaluated in large-scale multicenter studies. In addition, at 2022 American Society of Hematology (ASH) annual meeting, the preliminary results of a phase I clinical trial about a GPRC5D-targeted CAR-T cell product BMS-986393 in R/R MM patients were presented (NCT04674813). In this clinical trial, 10 patients who hadn’t received prior anti-BCMA therapy all achieved remission, and 7 patients who had failed in prior anti-BCMA therapy could also benefit from anti-GPRC5D CAR-T-cell therapy (28). Moreover, BCMA/GPRC5D bispecific CAR-T cell therapy is under active clinical exploration (NCT05431608). Additionally, the clinical trials of SLAMF7-targeted and integrin β7-targeted CAR-T cells are underway (NCT03778346). However, SLAMF7 and CCR10 are also expressed on activated T cells, which may result in CAR-T cell fratricide (21, 23).

Dual-targeted CAR-T cell therapy are also observed in preclinical and clinical studies, and they are available in a variety of forms, including combined infusion of 2 single‐targeted CAR-T cells, and application of bispecific CAR-T cells which incorporate two distinct scFvs into two CARs separately or a single CAR structure simultaneously, the latter also known as tandem CAR-T cells. In several clinical trials, CD38 and CD19 were applied in combination with BCMA to develop dual-targeted CAR-T cells for the treatment of R/R MM (26, 27, 30–33) (**Table 1**). In a phase I clinical trial, 23 R/R MM patients received BCMA/CD38 bispecific CAR-T cells, and 87% of them achieved clinical a response and 52% of them achieved complete response (CR) with a median follow-up of 9.0 months (30). In another clinical trial, 16 patients with R/R MM were treated with BCMA/CD38 bispecific CAR-T cells, and 14 of them had clinical response and 13 of them achieved CR after a median follow-up of 11.5 months (31). In addition to BCMA/CD38 bispecific CAR-T cells, the combination of anti-CD38 and anti-BCMA CAR-T cell therapy was also performed. In a phase 2, single-arm, single-center clinical trial, 22 patients with R/R MM received the combined infusion of the humanized anti-BCMA cells and the murine anti-CD38 CAR-T cells, and 91% of patients had clinical response and 55% of them achieved CR (25). In another single-arm phase II trial, 21 patients were treated with the combined infusion of anti-BCMA and anti-CD19 CAR-T cells, 20 of them achieved a clinical response and 3 of them achieved CR with a median follow-up of 179 days (26). With a longer follow-up, the number of patients enrolled in this trial was increased, and 62 R/R MM patients received the combined infusion of anti-BCMA and anti-CD19 CAR-T cells (26, 27). In this clinical trial, 92% of patients had a clinical response and 60% of them achieved CR with a median follow-up of 21.3 months (27). In addition, a recent study has explored the efficacy and safety of the combination of anti-CD19 and anti-BCMA CAR-T cell therapy in 10 newly diagnosed MM patients with high-risk factors, and all patients achieved a clinical response (32). In preclinical studies, BCMA/GPRC5D and BCMA/CS1 bispecific CAR-T cells showed robust anti-tumor activities against MM cells, and they could overcome BCMA-negative antigen escape (36, 37). Similarly, BCMA/CS1 bispecific CAR-T cells were also effective in R/R MM patients and able to prevent BCMA-negative relapse (34). Interestingly, some natural ligands are able to bind to two or more surface antigens on MM cells, so these CAR-T cells that are manufactured with the antigen-recognition domains derived from these ligands exhibit the ability to recognize several target antigens on malignant cells and achieve dual-antigen or multiple-antigen targeting. Several ligand-based CAR-T cells have been tested in preclinical studies and achieved satisfactory outcomes at present. For example, APRIL-based CAR-T cells could target both BCMA and TACI on MM cells (38), and BAFF ligand-based CAR-T cells could specifically recognize three different receptors on MM cells, including BAFF-R, BCMA, and TACI (39).

**Table 1:** Clinical trials of dual-targeted CAR-T cell therapy in MM.

Dual-targeted CAR-T cell therapy	Dose of CAR-T cells	No. of patients	Median follow-up	Response	Toxicities	Reference
**BCMA/CD38 bispecific CAR-T cells**	4.0 ×10^6^/kg	23 R/R MM patients (39% of them with EMD)	9 months	ORR 87%, sCR 52% PR 33%	CRS (87%), CRES (0%), cytopenia (96%), infections (22%)	Mei H et al. (30)
**BCMA/CD38 bispecific CAR-T cells**	median dose: 2.1 × 10^6^/kg (range: 0.5-10.0 × 10^6^/kg)	16 R/R MM patients (50% of them with EMD)	11.5 months	ORR 88%, CR 81%, PR 6%	CRS (75%), cytopenia (100%), HLH (6%), infections (38%)	Tang Y et al. (31)
**BCMA/CS1 bispecific CAR-T cells**	0.75 × 10^6^/kg, 1.5 × 10^6^/kg, 3.0 × 10^6^/kg	16 R/R MM patients(19% of them with EMD)	290 days	ORR 100%, sCR 31% PR 13%	CRS (38%) CRES (0%)	Li C et al. (34)
**Combined infusion of anti-BCMA and anti-CD38 CAR-T cells**	2 × 10^6^/kg, 2 × 10^6^/kg, respectively	22 R/R MM patients (14% of them with EMD)	24 months	ORR 91%, CR 55%,	CRS (100%), CRES (14%), cytopenia (100%) infections (17%)	Zhang H et al. (25)
**Combined infusion of anti-BCMA and anti-CD19 CAR-T cells**	1 × 10^6^/kg, 1 × 10^6^/kg, respectively	21 R/R MM patients	268 days	ORR 95%, CR 14%, PR 14% sCR 43%	CRS (90%), cytopenia(95%), B cell aplasia (100%), lung infections (5%)	Yan Z et al. (26)
**Combined infusion of anti-BCMA and anti-CD19 CAR-T cells**	1 × 10^6^/kg, 1 × 10^6^/kg, respectively	62 R/R MM patients (24% of them with EMD)	21.3 months	ORR 92%, CR 60%, PR 21%	CRS (95%), CRES (11%), cytopenia (98%), B cell aplasia (30%), infections (45%)	Wang Y et al. (27)
**Combined infusion of anti-BCMA and anti-CD19 CAR-T cells after auto-HSCT**	5 × 10^7^/kg, 1 × 10^7^/kg, respectively	10 high-risk NDMM patients	42 months	ORR 100%, CR 10% sCR 90%	CRS (100%), CRES (0%), cytopenia (100%), infections (100%)	Shi X et al. (35)
**Combined infusion of anti-BCMA and anti-CD19 CAR-T cells**	5 × 10^8^ cells, 5 × 10^8^ cells, respectively	10 MM patients with relapse (Phase A) and 20 high-risk MM patients (Phase B, as a randomized controlled trial)	follow-up ranging from 248 to 966 days in Phase B	ORR 23%, CR 6% PR 6%	CRS (90%), CRES (3%),	Garfall AL et al. (32)
**Combined infusion of** **anti-BCMA and anti-CD19 FasTCAR-T Cells**	1 × 10^5^/kg, 2 × 10^5^/kg, 3 × 10^5^/kg	13 high-risk NDMM patients	5.3 months	ORR 95% sCR 69%	CRS (23%) CRES (0%)	Du J et al. (33)

EMD, extramedullary disease; CRS, cytokine release syndrome; CRES, CAR-T-cell-related encephalopathy syndrome; HLH, hemophagocytic lymphohistiocytosis; auto-HSCT, autologous hematopoietic stem cell transplantation; NDMM, newly diagnosed multiple myeloma.

Besides targeting distinct antigens, increasing target antigen density on MM cells also appears to be an appealing strategy. Several studies have proved that γ-secretase inhibitors and all-trans retinoic acid (ATRA) could upregulate BCMA expression on MM cells and facilitate their recognition by anti-BCMA CAR-T cells (40, 41). In addition, ATRA could promote CD38 expression on MM cells (42).

### 2.2 Preventing CAR-T cell exhaustion

Short-term clinical remissions in R/R MM patients after anti-BCMA CAR-T cell therapy are partially attributed to CAR-T cell exhaustion, which is manifested as poor persistence and dysfunction of CAR-T cells. At present, it is considered that multiple factors are involved in CAR-T cell exhaustion, including persistent antigen stimulation and immunosuppressive tumor microenvironment, as well as the impaired function of T cells due to previous anti-myeloma therapy (43, 44). There are several potential strategies to ameliorate the dysfunction of CAR-T cells, such as optimizing CAR-T cell structure, utilizing early memory T cells (7, 45), and inhibiting intracellular exhaustion-related signals through genetic modifications or inhibitors. In addition, given the impaired cytotoxicity of T cells after multi-line anti-myeloma therapy, CAR-T cells manufacturing with T cells collected early in the disease course may be an effective strategy as well (43, 44).

#### 2.2.1 Optimizing CAR-T cell structure

At present, CD28, 4-1BB, ICOS, and OX40 are the most commonly used co-stimulatory molecules in CAR-T cell manufacturing. CD28 co-stimulation triggers robust T cell activation, so it could accelerate CAR-T cell exhaustion (46, 47). In contrast, 4-1BB co-stimulation is able to facilitate the expansion of stem cell memory T cells and ameliorate CAR-T cell exhaustion (48). ICOS is a member of the CD28 family, and the combination of ICOS and 4-1BB co-stimulation could remarkably increase the persistence of CAR-T cells (49). As a member of the TNF-R superfamily, OX40 exhibits the ability to promote T cell proliferation and memory formation. A recent study has proved that OX40-mediated BCMA-targeted CAR-T cells exhibited stronger proliferation ability and more durable anti-tumor activity under repeated BCMA stimulation compared with 4-1BB-mediated BCMA-targeted CAR-T cells (50). In addition, the fully humanized CAR structure could reduce immunogenicity of anti-BCMA CAR-T cells and avoid the immune-mediated rejection by the host immune system. More importantly, a phase 1 clinical trial has demonstrated that the R/R MM patients who had relapsed after prior murine-derived anti-BCMA CAR-T cell therapy could also achieve clinical responses from the fully humanized anti-BCMA CAR-T cells (7).

#### 2.2.2 Utilizing memory-phenotype CAR-T cells

Early memory T cells exhibit superior expansion and persistence. Similarly, a recent study reported that utilizing naïve or central memory T cells during CAR-T manufacturing processes could not only ameliorate CAR-T cell exhaustion, but also reduce the risk of severe cytokine release syndrome (51). In addition, it has been recently demonstrated that JQ1, an inhibitor of bromodomain and extra-terminal motif (BET) proteins, could maintain effector T cells with properties of central memory T cells and also enhance the persistence and function of adoptive CAR-T cells (52).

#### 2.2.3 Inhibiting exhaustion-related signals

BATF is a key factor involved in up-regulating a subset of exhaustion-related genes in CAR-T cells, and it has been demonstrated that depletion of BATF could enhance the anti-tumor activity of CAR-T cells and increase central memory CAR-T cells (53). Similarly, depletion of the endogenous TGF-β receptor II (TGFBR2) in CAR-T cells could not only prevent CAR-T cell exhaustion but also promote the formation of central memory CAR-T cells (54). Inhibiting intracellular calcium signaling and PD-1 signaling has also been shown to effectively prevent CAR-T cell exhaustion (55–57). In addition, the PI3K/AKT pathway is involved in T cell proliferation and differentiation, and it plays an important role in CAR-T cell exhaustion. At present, It has been confirmed that the PI3K inhibitor could modulate the differentiation of CAR-T cells and enhance the persistence of CAR-T cells *in vivo* (58–60). Intriguingly, a recent study found that the second generation tyrosine kinase inhibitor dasatinib could reverse the exhausted phenotype of CAR-T cells through increasing the expression of memory-associated genes, such as TCF7 and CCR7, and decreasing the expression of immune checkpoint molecule PD1 and exhaustion-related regulators, such as NR4A1, BATF3, ATF4, and FOS (61). Similarly, panobinosta also seems to have the potential to upregulate memory-associated genes and downregulate exhaustion-related genes (62). Moreover, a recent study demonstrated that SOX4 and ID3 are key exhaustion-related regulators, so inhibiting SOX4 and ID3 expression could also prevent CAR-T cell exhaustion (63).

#### 2.2.4 Improving CAR-T cell effector function

The exhausted phenotype of CAR-T cells exhibit impaired anti-tumor functionality. The anti-tumor activity of CAR-T cells can be improved through genetic modifications, including adding immune-stimulatory receptors and specifically deleting the genes mediated CAR-T cell anergy. At present, numerous studies have demonstrated that the armored CAR-T cells which secrete cytokines or express pro-inflammatory ligands, such as IL-7, IL-12, IL-15, IL-18, and CD40L, are able to reshape the tumor microenvironment (64–67). In addition, several studies have shown that additional chimeric co-stimulatory receptors (CCRs) could simultaneously enhance the killing effect of CAR-T cells and their persistence (68, 69). Additionally, another study has demonstrated that deletion of mediator complex subunit 12 and cyclin C in CAR-T cells could improve the anti-tumor activity of CAR-T cells (70, 71).

To improve the anti-tumor activity of CAR-T cells, combinatorial therapy with CAR-T cells and small molecule drugs, especially anti-myeloma agents, also seems to be a promising strategy. In clinical, lenalidomide has been used for the treatment of MM for a long time (72, 73). Interestingly, combination therapy with lenalidomide and CAR-T cells is able to achieve favorable outcomes and improve the cytotoxicity of CAR-T cells (74), and a case report showed that anti-BCMA CAR-T cells combined with lenalidomide were also effective in MM patients refractory to prior anti-BCMA CAR-T cell therapy (75). In addition, PD-1 blockade has been proven to enhance the killing activity of CAR-T cells against MM cells as well (37). However, CAR-T cell therapy in combination with small molecule drugs is still at a preliminary stage, and many combinational therapies are under investigation.

#### 2.2.5 Overcoming immunosuppressive tumor microenvironment

**Figure 2:**
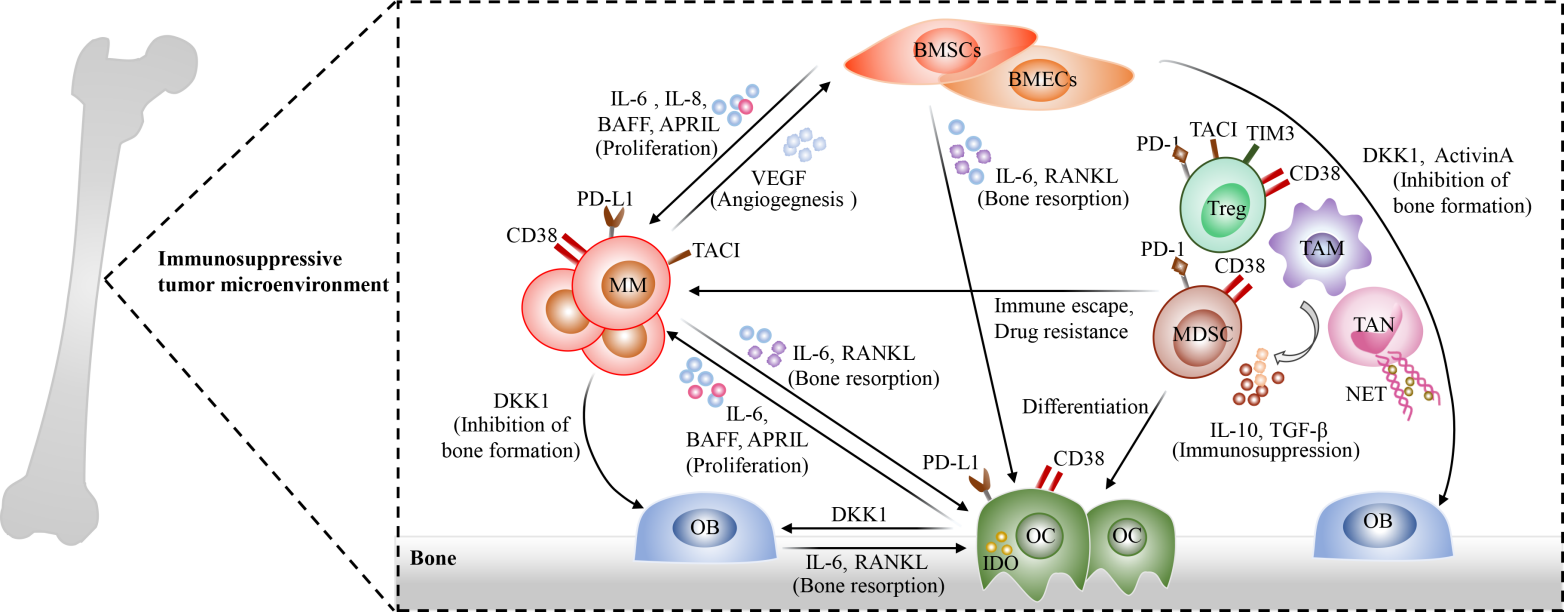
The complexity of bone marrow microenvironment in MM. Multiple immunosuppressive cells are accumulated in bone marrow microenvironment and exhibit tumor supportive properties, including osteoclasts (OCs)such as osteoclasts (OCs), myeloid-derived suppressor cells (MDSCs), regulatory T cells (Tregs), regulatory B cells(Bregs), tumor-associated macrophages (TAMs), tumor-associated neutrophils (TANs), and bone marrow stromal cells (BMSCs). These cells interact with surrounding MM cells through direct cell-to-cell contact or producing soluble factors, and then promote the proliferation, immune escape of MM cells as well as Drug resistance. Osteoclasts (OCs) are remarkably increased in the bone marrow microenvironment of MM patients and involved in the occurrence and development of myeloma bone disease. In addition, they produce APRIL, BAFF and IL-6 to promote MM cell proliferation and survival. Meanwhile, OCs act as antigen-presenting cells (APCs) in the bone marrow and exhibit immunosuppressive properties through up-regulating the expression of immune checkpoint molecules, such as PD-L1, CD38 and galectin 9. In turn, MM cells could promote bone resorption activity of OCs through the secretion of IL-6 and RANKL. Immunosuppressive Tregs and MDSCs, which express several immune inhibitory molecules, such as PD1, TIM3, and CD38, are significantly increased in MM bone marrow microenvironment and secrete TGF-β and IL-10 to promote the evasion of MM cells from immune surveillance. TAMs apparently infiltrate the bone marrow, and they promote angiogenesis and induce immune escape and drug resistance of MM cells. In addition, TANs also play an immunosuppressive role in MM bone marrow microenvironment through the release of neutrophil extracellular traps (NETs), which could contribute to tumor-associated thrombosis and tumor metastasis. Moreover, BMSCs show an inflammatory phenotype in MM microenvironment. On the one hand, they secrete several cytokines, such as APRIL, BAFF, IL-6, and RANKL; On the other hand, they induce the expression of anti-apoptotic proteins in MM cells, eventually promoting MM cell proliferation and drug resistance. Additionally, plasmacytoid dendritic cells (pDCs), NK cells, and NKT cells exhibit the decreased anti-myeloma activities in bone marrow microenvironment.

The bone marrow microenvironment of MM is complex, which is involved in promoting tumor growth, immune escape and drug resistance (76). There are multiple immunosuppressive cells accumulated in MM bone marrow microenvironment, which exhibit tumor supportive properties, such as osteoclasts (OCs), myeloid-derived suppressor cells (MDSCs), tumor-associated macrophages (TAMs), regulatory T cells (Tregs), regulatory B cells (Bregs), and tumor-associated neutrophils (TANs), as well as bone marrow stromal cells (BMSCs) (77–83). On the one hand, these cells crosstalk with MM cells and then promote the survival and proliferation of MM cells (78, 79, 84) (**Figure 2**). On the other hand, they impair the cytotoxicity of effector T cells through direct cell-to-cell contact or the release of soluble factors and then facilitate the evasion of MM cells from immune surveillance. OCs are multinucleated cells derived from hematopoietic stem cells and responsible for bone resorption, and they are significantly increased in the bone marrow and secrete RANKL, which are involved in the occurrence and development of myeloma bone disease. In addition, they release APRIL, BAFF and IL-6, which could promote the proliferation and survival of MM cells. More importantly, OCs also act as antigen-presenting cells (APCs) resident in the bone marrow and exert immunosuppressive functions through up-regulating the expression of immune checkpoint molecules, such as PD-L1, CD38 and galectin 9 (79). In turn, MM cells produce IL-6 and RANKL, which could enhance bone resorption activity of OCs. In MM patients, TAMs which display M2-like properties apparently infiltrate the bone marrow and exhibit robust activation of BAFF pro-proliferative signaling (85), and they are involved in promoting angiogenesis and tumor resistance (86, 87). In addition, massive MDSCs were accumulated in bone marrow microenvironment of MM patients (88). They could produce immunosuppressive molecules IL-10 and TGF-β, and then promote the generation of Treg cells and the immune escape of MM cells, as well as angiogenesis (89). Neutrophils are one of the important cell types in the bone marrow and known as the first line of defense against pathogens. They can form neutrophil extracellular traps (NETs) which play an important in defending against pathogens. However, NETs derived from TANs play an immunosuppressive role in MM bone marrow microenvironment (90, 91). A recent study has shown that MM cells are able to induce NET formation in a PAD4-depenedent manner, which is involved in promoting tumor-associated thrombosis and tumor metastasis (91). Furthermore, BMSCs exhibit an inflammatory phenotype with the activation of NF-κB signaling in MM microenvironment (92). They not only secrete several cytokines, such as APRIL, BAFF, IL-6, and RANKL, which play an important role in promoting MM cell proliferation, but also induce the expression of anti-apoptotic proteins in MM cells, including survivin and Mcl-1. Immunosuppressive Tregs and Bregs are also remarkably increased in MM bone marrow microenvironment, and they maintain immune tolerance through the secretion of immunosuppressive cytokines such as TGF-β and IL-10, as well as the expression of immune inhibitory molecules, such as PD1, TIM3, and CD38 (93). Moreover, NK cells exhibit an exhausted phenotype, mainly manifested as the decreased anti-myeloma activity with the downregulated expression of multiple activating receptors and cytolytic molecules, such as NKG2D, SLAMF7, CD69, and GZMA (94–96), and plasmacytoid dendritic cells (pDCs) are dysfunctional with an upregulated expression of PD-L1 (84). Besides, NKT cells are decreased in R/R MM patients (97).

**Figure 3:**
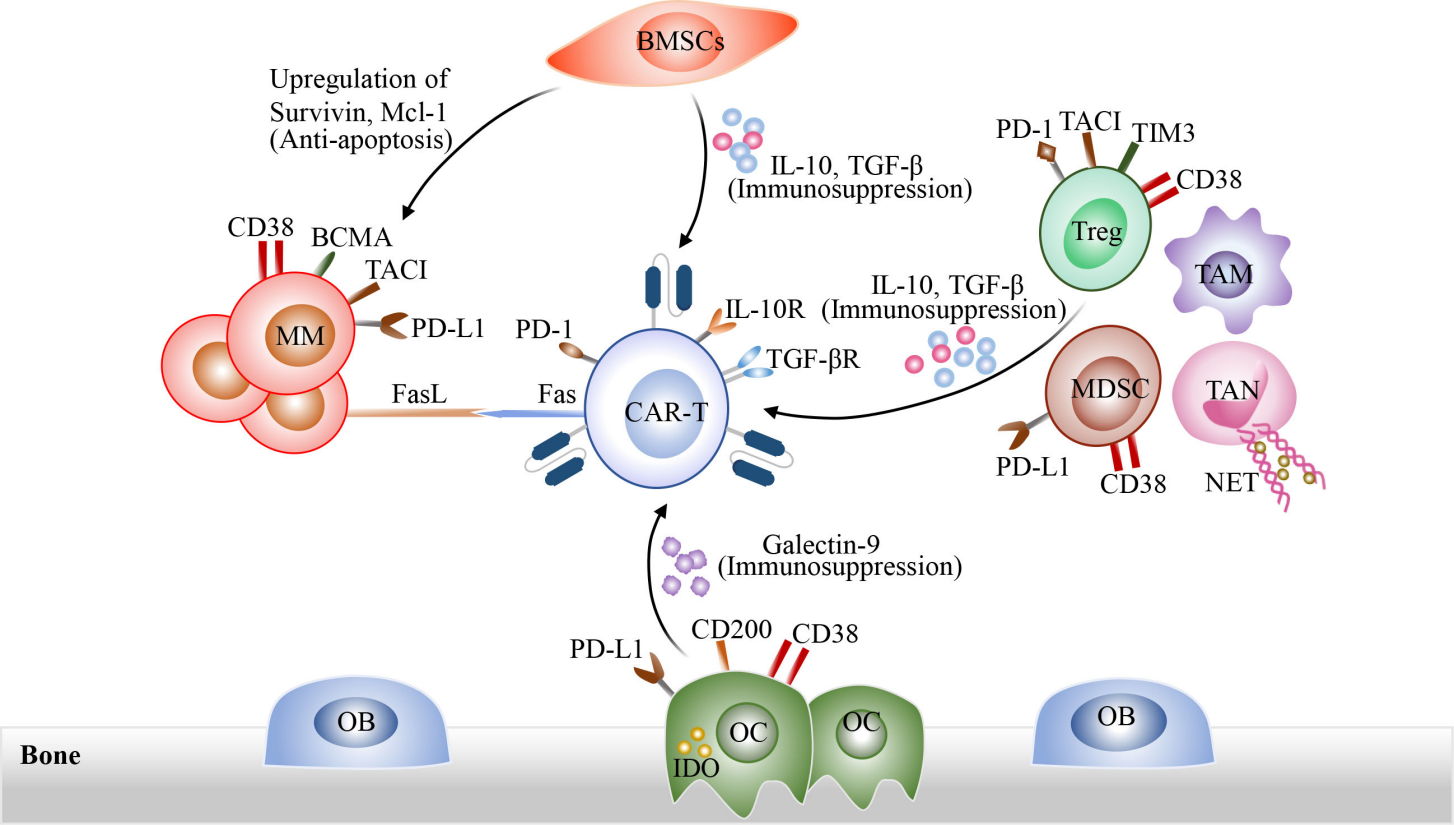
The interactions among CAR-T cells, tumor cells and immunosuppressive tumor microenvironment. On the one hand, tumor cells and immunosuppressive cells in bone marrow microenvironment induce CAR-T cell exhaustion through direct cell-to-cell contact, such as PD-1/PDL-1 pathway and Fas/FasL pathway. Immunosuppressive cells could also release immune inhibitory cytokines such as IL-10 and TGF-β to impact the cytotoxicity of CAR-T cells and promote the generation of Treg cells. In addition, BMSCs could protect MM cells against CAR-T cells through the up-regulation of anti-apoptosis proteins in MM cells.

Moreover, MM microenvironment, including MM cells, immunosuppressive cells, and BMSCs, as well as multiple soluble cytokines, interact with CAR-T cells, which could result in CAR-T cell dysfunction and inhibit engraftment of CAR-T cells, eventually promoting extrinsic resistance of MM cells after CAR-T cell infusion (85, 93, 98) (**Figure 3**). On the one hand, tumor cells and immunosuppressive cells in bone marrow microenvironment induce CAR-T cell exhaustion through direct cell-to-cell contact, such as PD-1/PDL-1 pathway and Fas/FasL pathway. On the other hand, immunosuppressive cells could also release immune inhibitory factors IL-10 and TGF-β to impair the cytotoxicity of CAR-T cells and promote the generation of Treg cells. In addition, BMSCs could protect MM cells against CAR-T Cells through upregulation of anti-apoptosis proteins in MM cells (99). Therefore, overcoming immunosuppressive tumor microenvironment may represent an promising therapeutic strategy. At present, the armored CAR-T cells which could release immune-activating cytokines *in situ* are developed to overcome hostile immunosuppressive tumor microenvironment (64–67). Due to CD38 expression on a variety of immune regulatory cells in MM bone marrow microenvironment, such as Tregs and MDSCs, anti-CD38 CAR-T cells also have a slight cytotoxicity against CD38 positive immune regulatory cells (100). CAR-T cell therapy combined with oncolytic viruses also appears to be a potential strategy to overcome immunosuppressive tumor microenvironment (101). Besides, CAR-T cell therapy in combination with FL118, an inhibitor of antiapoptotic proteins, has been proven to be able to overcome resistance induced by BMSCs (99).

## 3 Strategies to improve accessibility of CAR-T cell therapy

### 3.1 Developing universal CAR-T cell products

At present, all commercial CAR-T cell products are manufactured using autologous T lymphocytes. The personalized manufacturing processes approximately take 3-4 weeks and also result in high manufacturing costs. In particular, a portion of R/R MM patients suffer from rapid disease progression during CAR-T cell manufacturing, so they are unable to receive autologous CAR-T cell therapy in a timely fashion or even lose the opportunity to receive CAR-T cell therapy. The relatively longer manufacturing time and the higher manufacturing costs of autologous CAR-T cell products limit their accessibility, so the readily available “off the shelf “ allogeneic CAR-T cell products are currently being explored to overcome these limitations, such as universal CAR-T (UCAR-T) cells and CAR-γδ T cells (102). Because UCAR-T cells are derived from healthy donors, they exhibit several advantages, such as superior cytotoxicity and no malignant cell contamination. Moreover, due to the large-scale production of these UCAR-T cells, manufacturing costs are remarkably decreased. Unfortunately, these allogeneic UCAR-T cells might result in graft versus host disease (GVHD) and rejection by the host immune system (102). In a recent phase I clinical trial, 43 R/R MM patients were treated with allogeneic anti-BCMA CAR-T cells, and 55.8% of them showed a clinical response and 25% of them achieved a sCR with the median follow-up 10.2 months (103). More importantly, these allogeneic CAR-T cells were successfully administered with a median time from patient enrollment to CAR-T cell infusion of 5 days, which remarkably shortened the waiting time for CAR-T cell infusion. However, the overall response rate (ORR) of these allogeneic CAR-T cells is significantly lower than that of two FDA-approved anti-BCMA CAR-T cell products. In addition, γδ T cells can be utilized to generate UCAR-T cells. They are a small group of effector T cells with the expression of T cell receptors and natural killer receptors (NKRs). In particular, NKRs expressed on γδ T cells mediate tumor cell recognition in an MHC-independent manner (104–106). Thus, CAR-γδ T cells could simultaneously mediate both innate and adaptive anti-tumor immune responses *via* NKRs and CARs (107, 108). More importantly, γδ T cells did not induce GVHD in allogeneic hematopoietic stem cell transplantation (109). Furthermore, compared with CAR-T cells, CAR-γδ T cells significantly decrease cytokine production and show preferable efficacy. Currently, due to the widespread sources of NK cells and no induction of GVHD, CAR-NK cell therapy has also been regarded as a promising adoptive cell therapy and is being explored for the treatment of R/R MM in preclinical studies (110–112).

### 3.2 Bridging therapies

To prevent rapid disease progression during the manufacturing period and reduce baseline tumor burden, bridging therapies prior to CAR-T cell therapy are crucial. Bridging therapies are usually individualized according to prior treatment and disease characteristics of every patient. In general, bridging therapies with previously effective therapeutic agents can be considered, such as dexamethasone, daratumumab, carfilzomib, bortezomib, and pomalidomide (113). There are several bridging therapy options, such as chemotherapies, targeted therapies, autologous hematopoietic stem cell transplantation (auto-HSCT), and localized radiotherapy, as well as localized cryoablation. Given that BCMA-targeted agents may result in the decreased BCMA expression and then impact anti-BCMA CAR-T cell efficacy, they are often excluded from bridging therapies. Auto-HSCT is standard therapy for transplant-eligible MM patients, and it could also serve as an effective bridging therapy prior to CAR-T cell therapy (114). A recent study has demonstrated that auto-HSCT in combination with CAR-T cell therapy achieved higher ORR, PFS, and OS compared with CAR-T cell therapy alone, indicating that bridging auto-HSCT is able to promote durable and deep remission (115). Another clinical trial has compared the efficacy of the combination of auto-HSCT and CAR-T cell therapy with auto-HSCT alone, and it showed that the combination group had higher CR rate and 3-year PFS than the auto-HSCT group, with lower 3 year relapse rate (116). The above studies suggest that the combination of auto-HSCT and CAR-T cell therapy could exert a synergistic effect in remission induction (114, 116–118). In addition, localized radiotherapy and cryoablation are effective bridging therapies for R/R MM patients with bulky mass. Localized radiotherapy and cryoablation in combination with anti-BCMA CAR-T cell therapy may result in synergistic anti-tumor effect (119, 120). On the one hand, radiotherapy and cryoablation could directly destroy tumor cells; On the other hand, they could sensitize CAR-T cells and activate endogenous effector T cells through the abscopal effect, which may be associated with the upregulation of intratumoral chemokines and cytokines and the release of neo-antigens (119–121). In particular, radiotherapy could also activate CAR-T cells through immunogenic cell death (122).

### 3.3 Employing rapid CAR-T cell manufacturing platform

Rapid CAR-T cell manufacturing can also shorten the interval between patient enrollment to CAR-T cell infusion. Encouragingly, it has been reported that the FasT CAR-T cells, which were manufactured the next day and underwent approximately 7 days of quality control testing, showed favorable efficacy in B-cell acute lymphoblastic leukemia in preclinical and clinical studies (123, 124). Due to the remarkably shortened manufacturing time, they are more suitable for patients with progressive disease and able to decrease patients’ clinical hospital stays, eventually improving the accessibility of CAR-T cell therapy. In addition, due to the short-term culture *in vitro*, FasT CAR-T cells show a less exhausted phenotype and superior killing activities compared with conventional CAR-T cells (123, 124). The 2022 ASH meeting announced a phase I study of BCMA/CD19 dual-targeted FasT CAR-T cells (GC012F) in NDMM patients (NCT04935580), and these FasT CAR-T cells were prepared in 22 to 36 hours (33). In addition, another ongoing study about BCMA Nex T CAR-T cell therapy BMS-986354 in R/R MM patients were also mentioned in the 2022 ASH meeting (NCT04394650). In this phase I clinical trial, these CAR-T cells were manufactured within 5 to 6 days using the NEX-T process and showed potent killing potency (125). However, the efficacy of these CAR-T cells remains to be determined in more studies.

### 3.4 Utilizing non-viral transfection

In addition, non-viral transfection could also reduce the manufacturing costs and increase the accessibility of CAR-T cell therapy. Transduction of CAR genes into T cells is a vital step in CAR-T cell manufacturing processes. Currently, CAR transfection is frequently achieved by viral vectors, such as gammaretroviral and lentiviral vectors. However, the production of viral vectors usually takes two to three weeks and requires good manufacturing practice (cGMP)-grade facilities and trained operators, which makes CAR-T cell manufacturing time-consuming and expensive. In addition, transduced sequences *via* viral vectors are limited. Therefore, virus-free genetic modification methods are being actively explored. At present, transposon systems, including piggyBac (PB) and Sleeping Beauty (SB) systems, have showed stable gene transfer efficiency in CAR-T cell manufacturing in preclinical and clinical studies (126–129). Due to the decreased complexity of manufacturing processes and the better cargo capacity, transposon systems reduce the manufacturing costs and are more suitable for multi-targeted CAR-T cell manufacturing compared with viral vectors. Furthermore, transposon systems can be utilized on an automated process platform to generate clinical therapeutic doses of CAR-T cells, which will further promote the scale-up manufacturing of CAR-T cells and increase R/R patient access to CAR-T cell therapy (128, 130). In addition, transposon-based CAR-T cells exhibit early memory T cell phenotype (128). Encouragingly, a recent study demonstrated that CRISPR-Cas9-mediated non-viral specifically targeted CAR-T cells were safe and effective in patients with R/R non-Hodgkin lymphoma (NHL) (56), indicating that CRISPR-Cas9 is a new tool for precise genome editing in CAR-T cell manufacturing and will facilitate the development of more gene-specific targeted CAR-T cells in the future (56, 131).

### 3.5 Initiating CAR-T cell therapy in earlier lines of treatment for high-risk MM patients

High-risk newly diagnosed MM (NDMM) patients usually have poor prognosis with standard first-line therapy, so there is a significant unmet need in additional therapeutic options for these high-risk MM patients. It seems that CAR-T cell therapy may provide a potential solution and serve as first-line therapy for these high-risk NDMM patients. The 2022 ASH meeting reported an ongoing multicenter study about BCMA/CD19 dual-targeted FasT CAR-T cells in NDMM patients (NCT04935580). In this clinical trial, 13 high-risk NDMM patients were treated with BCMA/CD19 dual-targeted FasT CAR-T cells, and 100% of them achieved a clinical response and 69% of them achieved a sCR after a median follow-up 5.3 months (33). These results revealed that CAR-T cell therapy in earlier lines of treatment is safe and may induce deep responses in high-risk MM patients, eventually increasing their accessibility for high-risk MM patients.

## 4 Subsequent anti-myeloma therapy after CAR-T therapy

At present, relapses occur frequently after anti-BCMA CAR-T cell therapy, especially in high-risk MM patients (132–134). However, currently there is a lack of recommended salvage treatment for R/R MM patients after relapse on CAR-T cell therapy. Therefore, there is an urgent need to explore suitable subsequent therapy for R/R MM patients who have been refractory to anti-BCMA CAR-T cell therapy. In addition to treatment with optimized CAR-T therapy and previous chemotherapy regimens, as well as auto-HSCT (134, 135), novel anti-myeloma agents provide additional salvage options for R/R MM patients who have relapsed after anti-BCMA CAR-T cell therapy, including selinexor, carfilzomib, pomalidomide, monoclonal antibodies, and T cell redirecting bispecific antibodies (132–134, 136). Furthermore, several studies showed that the R/R MM patients who had experienced relapse after anti-BCMA CAR-T cell therapy could also benefit from carfilzomib-based therapy, venetoclax-based therapy, and selinexor-based therapy (132–134, 136). In addition, T cell redirecting bispecific antibodies, such as Cevostamab and Talquetamab, have also proved to be feasible salvage treatment after anti-BCMA CAR-T cell therapy and able to induce durable responses (137–139). Moreover, a phospholipid-drug complex Iopofosine I-131 could also achieve clinical responses in R/R MM patients who had failed in prior anti-BCMA therapy (140).

In addition, there are few recommendations for maintenance/consolidation therapy after CAR-T cell infusion, but it seems that maintenance treatment after CAR-T therapy may provide potential clinical benefits for high-risk MM patients. Recent studies have demonstrated that maintenance therapy with lenalidomide and pomalidomide is able to facilitate CAR-T cell re-expansion in high-risk MM patients (32, 35). Moreover, in a phase I study, the efficacy and safety of selinexor in R/R MM patients with EMD after the fully humanized anti-BCMA CAR-T therapy is being tested (NCT05201118).

## 5 Conclusion

In recent years, anti-BCMA CAR-T cell therapy has achieved impressive outcomes in in R/R MM and its side effects are generally controllable, but there are still several challenges to be addressed. For example, relapse continues to occur after anti-BCMA CAR-T cell therapy, and high manufacturing costs and the longer manufacturing cycle of autologous CAR-T cell products limit their accessibility. Thus, further improvement is required. At present, potential mechanisms and therapeutic strategies are being explored, such as identification of novel therapeutic targets, optimization of CAR structure and genetic modification methods, application of dual-targeted CAR-T cell therapy, and combination of CAR-T cell therapy with other approaches. However, due to the resistance to CAR-T cell therapy and persistent high-risk factors, subsequent anti-myeloma therapy is also of great clinical significance.

## Author contributions

YX and XZ designed the manuscript. XZ, HZ, HL, and JW drafted the manuscript and created the figures. XZ and YX revised the manuscript. All authors contributed to the article and approved the submitted version.

## References

[1] Facon T Kumar S Plesner T Orlowski RZ Moreau P Bahlis N. Daratumumab plus lenalidomide and dexamethasone for untreated myeloma. N Engl J Med (2019) 380(22):2104–15. doi: 10.1056/NEJMoa1817249 PMC10045721 31141632

[2] Misund K Hofste Op Bruinink D Coward E Hoogenboezem RM Rustad EH Sanders MA. Clonal evolution after treatment pressure in multiple myeloma: Heterogenous genomic aberrations and transcriptomic convergence. Leukemia (2022) 36(7):1887–97. doi: 10.1038/s41375-022-01597-y PMC9252918 35643867

[3] Xu J Chen LJ Yang SS Sun Y Wu W Liu YF. Exploratory trial of a biepitopic CAR T-targeting b cell maturation antigen in relapsed/refractory multiple myeloma. Proc Natl Acad Sci U.S.A. (2019) 116(19):9543–51. doi: 10.1073/pnas.1819745116 PMC6510991 30988175

[4] Zhao WH Liu J Wang BY Chen YX Cao XM Yang Y. A phase 1, open-label study of LCAR-B38M, a chimeric antigen receptor T cell therapy directed against b cell maturation antigen, in patients with relapsed or refractory multiple myeloma. J Hematol Oncol (2018) 11(1):141. doi: 10.1186/s13045-018-0681-6 30572922 PMC6302465

[5] Raje N Berdeja J Lin Y Siegel D Jagannath S Madduri D. Anti-BCMA CAR T-cell therapy bb2121 in relapsed or refractory multiple myeloma. N Engl J Med (2019) 380(18):1726–37. doi: 10.1056/NEJMoa1817226 PMC8202968 31042825

[6] Cohen AD Garfall AL Stadtmauer EA Melenhorst JJ Lacey SF Lancaster E. B cell maturation antigen-specific CAR T cells are clinically active in multiple myeloma. J Clin Invest (2019) 129(6):2210–21. doi: 10.1172/JCI126397 PMC6546468 30896447

[7] Wang D Wang J Hu G Wang W Xiao Y Cai H. A phase 1 study of a novel fully human BCMA-targeting CAR (CT103A) in patients with relapsed/refractory multiple myeloma. Blood (2021) 137(21):2890–901. doi: 10.1182/blood.2020008936 33512480

[8] Deng H Liu M Yuan T Zhang H Cui R Li J. Efficacy of humanized anti-BCMA CAR T cell therapy in Relapsed/Refractory multiple myeloma patients with and without extramedullary disease. Front Immunol (2021) 12:720571. doi: 10.3389/fimmu.2021.720571 34421924 PMC8374046

[9] Que Y Xu M Xu Y Almeida VDF Zhu L Wang Z. Anti-BCMA CAR-T cell therapy in Relapsed/Refractory multiple myeloma patients with extramedullary disease: A single center analysis of two clinical trials. Front Immunol (2021) 12:755866. doi: 10.3389/fimmu.2021.755866 34777368 PMC8589080

[10] Zhou D Wang Y Cheng H Zhu L Chen W Li H. Factors associated with infection events after chimeric antigen receptor T-cell therapy for relapsed or refractory multiple myeloma. J Infect Chemother (2022) 29(2):179–85. doi: 10.1016/j.jiac.2022.10.012 36368473

[11] Wang Y Li C Xia J Li P Cao J Pan B. Humoral immune reconstitution after anti-BCMA CAR T-cell therapy in relapsed/refractory multiple myeloma. Blood Adv (2021) 5(23):5290–9. doi: 10.1182/bloodadvances.2021004603 PMC9153033 34587230

[12] Logue JM Peres LC Hashmi H Colin-Leitzinger C Shrewsbury AM Hosoya H. Early cytopenias and infections after standard of care idecabtagene vicleucel in relapsed or refractory multiple myeloma. Blood Adv (2022) 6(24):6109–19. doi: 10.1182/bloodadvances.2022008320 PMC9768247 35939783

[13] Berdeja JG Madduri D Usmani SZ Jakubowiak A Agha M Cohen AD. Ciltacabtagene autoleucel, a b-cell maturation antigen-directed chimeric antigen receptor T-cell therapy in patients with relapsed or refractory multiple myeloma (CARTITUDE-1): a phase 1b/2 open-label study. Lancet (2021) 398(10297):314–24. doi: 10.1016/S0140-6736(21)00933-8 34175021

[14] Cliff ERS Mian H Mohyuddin GR. Teclistamab in relapsed or refractory multiple myeloma. N Engl J Med (2022) 387(18):1721–2. doi: 10.1056/NEJMc2211969 36322860

[15] Gazeau N Beauvais D Yakoub-Agha I Mitra S Campbell TB Facon T. Effective anti-BCMA retreatment in multiple myeloma. Blood Adv (2021) 5(15):3016–20. doi: 10.1182/bloodadvances.2021004176 PMC8361465 34351389

[16] Zhou X Rasche L Kortüm KM Mersi J Einsele H. BCMA loss in the epoch of novel immunotherapy for multiple myeloma: From biology to clinical practice. Haematologica (2022). doi: 10.3324/haematol.2020.266841 PMC10071122 36263838

[17] Samur MK Fulciniti M Aktas Samur A Bazarbachi AH Tai YT Prabhala R. Biallelic loss of BCMA as a resistance mechanism to CAR T cell therapy in a patient with multiple myeloma. Nat Commun (2021) 12(1):868. doi: 10.1038/s41467-021-21177-5 33558511 PMC7870932

[18] Radhakrishnan SV Luetkens T Scherer SD Davis P Vander Mause ER Olson ML. CD229 CAR T cells eliminate multiple myeloma and tumor propagating cells without fratricide. Nat Commun (2020) 11(1):798. doi: 10.1038/s41467-020-14619-z 32034142 PMC7005855

[19] Murad JM Baumeister SH Werner L Daley H Trébéden-Negre H Reder J. Manufacturing development and clinical production of NKG2D chimeric antigen receptor-expressing T cells for autologous adoptive cell therapy. Cytotherapy (2018) 20(7):952–63. doi: 10.1016/j.jcyt.2018.05.001 PMC6127861 30180944

[20] Casucci M Nicolis di Robilant B Falcone L Camisa B Norelli M Genovese P. CD44v6-targeted T cells mediate potent antitumor effects against acute myeloid leukemia and multiple myeloma. Blood (2013) 122(20):3461–72. doi: 10.1182/blood-2013-04-493361 24016461

[21] O’Neal J Ritchey JK Cooper ML Niswonger J Sofía González L Street E. CS1 CAR-T targeting the distal domain of CS1 (SLAMF7) shows efficacy in high tumor burden myeloma model despite fratricide of CD8+CS1 expressing CAR-T cells. Leukemia (2022) 36(6):1625–34. doi: 10.1038/s41375-022-01559-4 PMC9162922 35422095

[22] Hosen N Matsunaga Y Hasegawa K Matsuno H Nakamura Y Makita M. The activated conformation of integrin β_7_ is a novel multiple myeloma-specific target for CAR T cell therapy. Nat Med (2017) 23(12):1436–43. doi: 10.1038/nm.4431 29106400

[23] Ferguson ID Patiño-Escobar B Tuomivaara ST Lin YT Nix MA Leung KK. The surfaceome of multiple myeloma cells suggests potential immunotherapeutic strategies and protein markers of drug resistance. Nat Commun (2022) 13(1):4121. doi: 10.1038/s41467-022-31810-6 35840578 PMC9287322

[24] Mailankody S Devlin SM Landa J Nath K Diamonte C Carstens EJ. GPRC5D-targeted CAR T cells for myeloma. N Engl J Med (2022) 387(13):1196–206. doi: 10.1056/NEJMoa2209900 PMC10309537 36170501

[25] Zhang H Liu M Xiao X Lv H Jiang Y Li X. A combination of humanized anti-BCMA and murine anti-CD38 CAR-T cell therapy in patients with relapsed or refractory multiple myeloma. Leuk Lymphoma (2022) 63(6):1418–27. doi: 10.1080/10428194.2022.2030476 35105265

[26] Yan Z Cao J Cheng H Qiao J Zhang H Wang Y. A combination of humanised anti-CD19 and anti-BCMA CAR T cells in patients with relapsed or refractory multiple myeloma: a single-arm, phase 2 trial. Lancet Haematol (2019) 6(10):e521–9. doi: 10.1016/S2352-3026(19)30115-2 31378662

[27] Wang Y Cao J Gu W Shi M Lan J Yan Z. Long-term follow-up of combination of b-cell maturation antigen and CD19 chimeric antigen receptor T cells in multiple myeloma. J Clin Oncol (2022) 40(20):2246–56. doi: 10.1200/JCO.21.01676 35333600

[28] Bal S Kocoglu MH Nadeem O Htut M Gregory T Anderson LD Jr. Clinical activity of BMS-986393 (CC-95266), a G protein-coupled receptor class c group 5 member d (GPRC5D)-targeted chimeric antigen receptor (CAR) T cell therapy, in patients with relapsed and/or refractory (R/R) multiple myeloma (MM): First results from a phase 1, multicenter, open-label study. Blood (2022) 140(Supplement 1):883–5. doi: 10.1182/blood-2022-162395

[29] Zhang M Wei G Zhou L Zhou J Chen S Zhang W. GPRC5D CAR T cells (OriCAR-017) in patients with relapsed or refractory multiple myeloma (POLARIS): a first-in-human, single-centre, single-arm, phase 1 trial. Lancet Haematol (2023) 10(2):e107–16. doi: 10.1016/S2352-3026(22)00372-6 36725117

[30] Mei H Li C Jiang H Zhao X Huang Z Jin D. A bispecific CAR-T cell therapy targeting BCMA and CD38 in relapsed or refractory multiple myeloma. J Hematol Oncol (2021) 14(1):161. doi: 10.1186/s13045-021-01170-7 34627333 PMC8501733

[31] Tang Y Yin H Zhao X Jin D Liang Y Xiong T. High efficacy and safety of CD38 and BCMA bispecific CAR-T in relapsed or refractory multiple myeloma. J Exp Clin Cancer Res (2022) 41(1):2. doi: 10.1186/s13046-021-02214-z 34980210 PMC8722124

[32] Garfall AL Cohen AD Susanibar-Adaniya SP Hwang WT Vogl DT Waxman AJ. Anti-BCMA/CD19 CAR T cells with early immunomodulatory maintenance for multiple myeloma responding to initial or later-line therapy. Blood Cancer Discovery (2022). doi: 10.1158/2643-3230.BCD-22-0074. PMC9975770 36413381

[33] Du J Fu W Lu J Qiang W He H Liu J. Phase I open-label single-arm study of BCMA/CD19 dual-targeting FasT CAR-T cells (GC012F) as first-line therapy for transplant-eligible newly diagnosed high-risk multiple myeloma. Blood (2022) 140:889–90. doi: 10.1182/blood-2022-162295

[34] Li C Wang X Wu Z Luo W Zhang Y Kang Y. Bispecific CS1-BCMA CAR-T cells are clinically active in relapsed or refractory multiple myeloma: An updated clinical study. Blood (2022) 140(Supplement 1):4573–4. doi: 10.1182/blood-2022-170686 PMC10776387 37848634

[35] Shi X Yan L Shang J Kang L Yan Z Jin S. Anti-CD19 and anti-BCMA CAR T cell therapy followed by lenalidomide maintenance after autologous stem-cell transplantation for high-risk newly diagnosed multiple myeloma. Am J Hematol (2022) 97(5):537–47. doi: 10.1002/ajh.26486 35114022

[36] Fernández de Larrea C Staehr M Lopez AV Ng KY Chen Y Godfrey WD. Defining an optimal dual-targeted CAR T-cell therapy approach simultaneously targeting BCMA and GPRC5D to prevent bcma escape-driven relapse in multiple myeloma. Blood Cancer Discovery (2020) 1(2):146–54. doi: 10.1158/2643-3230.BCD-20-0020 PMC7575057 33089218

[37] Zah E Nam E Bhuvan V Tran U Ji BY Gosliner SB. Systematically optimized BCMA/CS1 bispecific CAR-T cells robustly control heterogeneous multiple myeloma. Nat Commun (2020) 11(1):2283. doi: 10.1038/s41467-020-16160-5 32385241 PMC7210316

[38] Lee L Draper B Chaplin N Philip B Chin M Galas-Filipowicz D. An APRIL-based chimeric antigen receptor for dual targeting of BCMA and TACI in multiple myeloma. Blood (2018) 131(7):746–58. doi: 10.1182/blood-2017-05-781351 PMC5922275 29284597

[39] Wong DP Roy NK Zhang K Anukanth A Asthana A Shirkey-Son NJ. A BAFF ligand-based CAR-T cell targeting three receptors and multiple b cell cancers. Nat Commun (2022) 13(1):217. doi: 10.1038/s41467-021-27853-w 35017485 PMC8752722

[40] Pont MJ Hill T Cole GO Abbott JJ Kelliher J Salter AI. Gamma-secretase inhibition increases efficacy of BCMA-specific chimeric antigen receptor T cells in multiple myeloma. Blood (2019) 134(19):1585–97. doi: 10.1182/blood.2019000050 PMC6871311 31558469

[41] García-Guerrero E Rodríguez-Lobato LG Sierro-Martínez B Danhof S Bates S Frenz S. ATRA works synergistically with the γ-secretase inhibitor crenigacestat to augment BCMA on multiple myeloma and the efficacy of BCMA-CAR T-cells. Haematologica (2023) 108(2):568–80. doi: 10.3324/haematol.2021.281339 PMC9890012 36722406

[42] Nijhof IS Groen RW Lokhorst HM van Kessel B Bloem AC van Velzen J. Upregulation of CD38 expression on multiple myeloma cells by all-trans retinoic acid improves the efficacy of daratumumab. Leukemia (2015) 29(10):2039–49. doi: 10.1038/leu.2015.123 25975191

[43] Mika T Ladigan-Badura S Maghnouj A Mustafa B Klein-Scory S Baraniskin A. Altered T-lymphocyte biology following high-dose melphalan and autologous stem cell transplantation with implications for adoptive T-cell therapy. Front Oncol (2020) 10:568056. doi: 10.3389/fonc.2020.568056 33363008 PMC7759611

[44] Abecassis A Roders N Fayon M Choisy C Nelson E Harel S. CAR-T cells derived from multiple myeloma patients at diagnosis have improved cytotoxic functions compared to those produced at relapse or following daratumumab treatment. EJHaem (2022) 3(3):970–4. doi: 10.1002/jha2.479 PMC9421998 36051036

[45] McLellan AD Ali Hosseini Rad SM. Chimeric antigen receptor T cell persistence and memory cell formation. Immunol Cell Biol (2019) 97(7):664–74. doi: 10.1111/imcb.12254 31009109

[46] Zhao Z Condomines M van der Stegen SJC Perna F Kloss CC Gunset G. Structural design of engineered costimulation determines tumor rejection kinetics and persistence of CAR T cells. Cancer Cell (2015) 28(4):415–28. doi: 10.1016/j.ccell.2015.09.004 PMC5003056 26461090

[47] Guedan S Madar A Casado-Medrano V Shaw C Wing A Liu F. Single residue in CD28-costimulated CAR-T cells limits long-term persistence and antitumor durability. J Clin Invest (2020) 130(6):3087–97. doi: 10.1172/JCI133215 PMC7260017 32069268

[48] Long AH Haso WM Shern JF Wanhainen KM Murgai M Ingaramo M. 4-1BB costimulation ameliorates T cell exhaustion induced by tonic signaling of chimeric antigen receptors. Nat Med (2015) 21(6):581–90. doi: 10.1038/nm.3838 PMC4458184 25939063

[49] Guedan S Posey AD Jr Shaw C Wing A Da T Patel PR. Enhancing CAR T cell persistence through ICOS and 4-1BB costimulation. JCI Insight (2018) 3(1):e96976. doi: 10.1172/jci.insight.96976 29321369 PMC5821198

[50] Tan J Jia Y Zhou M Fu C Tuhin IJ Ye J. Chimeric antigen receptors containing the OX40 signalling domain enhance the persistence of T cells even under repeated stimulation with multiple myeloma target cells. J Hematol Oncol (2022) 15(1):39. doi: 10.1186/s13045-022-01244-0 35365211 PMC8974082

[51] Arcangeli S Bove C Mezzanotte C Camisa B Falcone L Manfredi F. CAR T cell manufacturing from naive/stem memory T lymphocytes enhances antitumor responses while curtailing cytokine release syndrome. J Clin Invest (2022) 132(12):e150807. doi: 10.1172/JCI150807 35503659 PMC9197529

[52] Kagoya Y Nakatsugawa M Yamashita Y Ochi T Guo T Anczurowski M. BET bromodomain inhibition enhances T cell persistence and function in adoptive immunotherapy models. J Clin Invest (2016) 126(9):3479–94. doi: 10.1172/JCI86437 PMC5004946 27548527

[53] Zhang X Zhang C Qiao M Cheng C Tang N Lu S. Depletion of BATF in CAR-T cells enhances antitumor activity by inducing resistance against exhaustion and formation of central memory cells. Cancer Cell (2022) 40(11):1407–1422.e7. doi: 10.1016/j.ccell.2022.09.013 36240777

[54] Tang N Cheng C Zhang X Qiao M Li N Mu W. TGF-β inhibition *via* CRISPR promotes the long-term efficacy of CAR T cells against solid tumors. JCI Insight (2020) 5(4):e133977. doi: 10.1172/jci.insight.133977 31999649 PMC7101140

[55] Liu X Zhang Y Li K Liu Y Xu J Ma J. A novel dominant-negative PD-1 armored anti-CD19 CAR T cell is safe and effective against refractory/relapsed b cell lymphoma. Transl Oncol (2021) 14(7):101085. doi: 10.1016/j.tranon.2021.101085 33813229 PMC8050776

[56] Zhang J Hu Y Yang J Li W Zhang M Wang Q. Non-viral, specifically targeted CAR-T cells achieve high safety and efficacy in b-NHL. Nature (2022) 609(7926):369–74. doi: 10.1038/s41586-022-05140-y PMC9452296 36045296

[57] Shao M Teng X Guo X Zhang H Huang Y Cui J. Inhibition of calcium signaling prevents exhaustion and enhances anti-leukemia efficacy of CAR-T cells *via* SOCE-Calcineurin-NFAT and glycolysis pathways. Adv Sci (Weinh) (2022) 9(9):e2103508. doi: 10.1002/advs.202103508 35032108 PMC8948559

[58] Zheng W O’Hear CE Alli R Basham JH Abdelsamed HA Palmer LE. PI3K orchestration of the *in vivo* persistence of chimeric antigen receptor-modified T cells. Leukemia (2018) 32(5):1157–67. doi: 10.1038/s41375-017-0008-6 PMC5943191 29479065

[59] Wei C Xia K Xie Y Ye S Ding Y Liu Z. Combination of 4-1BB and DAP10 promotes proliferation and persistence of NKG2D(bbz) CAR-T cells. Front Oncol (2022) 12:893124. doi: 10.3389/fonc.2022.893124 35965586 PMC9372572

[60] Funk CR Wang S Chen KZ Waller A Sharma A Edgar CL. PI3Kδ/γ inhibition promotes human CART cell epigenetic and metabolic reprogramming to enhance antitumor cytotoxicity. Blood (2022) 139(4):523–37. doi: 10.1182/blood.2021011597 PMC8796652 35084470

[61] Zhang H Hu Y Shao M Teng X Jiang P Wang X. Dasatinib enhances anti-leukemia efficacy of chimeric antigen receptor T cells by inhibiting cell differentiation and exhaustion. J Hematol Oncol (2021) 14(1):113. doi: 10.1186/s13045-021-01117-y 34289897 PMC8293573

[62] Ali AI Wang M von Scheidt B Dominguez PM Harrison AJ Tantalo DGM. A histone deacetylase inhibitor, panobinostat, enhances chimeric antigen receptor T-cell antitumor effect against pancreatic cancer. Clin Cancer Res (2021) 27(22):6222–34. doi: 10.1158/1078-0432.CCR-21-1141.s 34475103

[63] Good CR Aznar MA Kuramitsu S Samareh P Agarwal S Donahue G. An NK-like CAR T cell transition in CAR T cell dysfunction. Cell (2021) 184(25):6081–6100.e26. doi: 10.1016/j.cell.2021.11.016 34861191 PMC8827167

[64] Yeku OO Brentjens RJ. Armored CAR T-cells: Utilizing cytokines and pro-inflammatory ligands to enhance CAR T-cell anti-tumour efficacy. Biochem Soc Trans (2016) 44(2):412–8. doi: 10.1042/BST20150291 PMC5529098 27068948

[65] Lanitis E Rota G Kosti P Ronet C Spill A Seijo B. Optimized gene engineering of murine CAR-T cells reveals the beneficial effects of IL-15 coexpression. J Exp Med (2021) 218(2):e20192203. doi: 10.1084/jem.20192203 33156338 PMC7653685

[66] Shum T Omer B Tashiro H Kruse RL Wagner DL Parikh K. Constitutive signaling from an engineered IL7 receptor promotes durable tumor elimination by tumor-redirected T cells. Cancer Discovery (2017) 7(11):1238–47. doi: 10.1158/2159-8290.CD-17-0538 PMC5669830 28830878

[67] Kuhn NF Purdon TJ van Leeuwen DG Lopez AV Curran KJ Daniyan AF. CD40 ligand-modified chimeric antigen receptor T cells enhance antitumor function by eliciting an endogenous antitumor response. Cancer Cell (2019) 35(3):473–488.e6. doi: 10.1016/j.ccell.2019.02.006 30889381 PMC6428219

[68] Katsarou A Sjöstrand M Naik J Mansilla-Soto J Kefala D Kladis G. Combining a CAR and a chimeric costimulatory receptor enhances T cell sensitivity to low antigen density and promotes persistence. Sci Transl Med (2021) 13(623):eabh1962. doi: 10.1126/scitranslmed.abh1962 34878825 PMC9869449

[69] Liao Q Mao Y He H Ding X Zhang X Xu J. PD-L1 chimeric costimulatory receptor improves the efficacy of CAR-T cells for PD-L1-positive solid tumors and reduces toxicity. vivo. biomark Res (2020) 8(1):57. doi: 10.1186/s40364-020-00237-w 33292688 PMC7607631

[70] Freitas KA Belk JA Sotillo E Quinn PJ Ramello MC Malipatlolla M. Enhanced T cell effector activity by targeting the mediator kinase module. Science (2022) 378(6620):eabn5647. doi: 10.1126/science.abn5647 36356142 PMC10335827

[71] Zebley CC Youngblood B. Improving antitumor T cells. Science (2022) 378(6620):598. doi: 10.1126/science.adf0546 36356156

[72] Syed YY. Selinexor: First global approval. Drugs (2019) 79(13):1485–94. doi: 10.1007/s40265-019-01188-9 31429063

[73] Usmani SZ Quach H Mateos MV Landgren O Leleu X Siegel D. Carfilzomib, dexamethasone, and daratumumab versus carfilzomib and dexamethasone for patients with relapsed or refractory multiple myeloma (CANDOR): updated outcomes from a randomised, multicentre, open-label, phase 3 study. Lancet Oncol (2022) 23(1):65–76. doi: 10.1016/S1470-2045(21)00579-9 34871550

[74] Works M Soni N Hauskins C Sierra C Baturevych A Jones JC. Anti-b-cell maturation antigen chimeric antigen receptor T cell function against multiple myeloma is enhanced in the presence of lenalidomide. Mol Cancer Ther (2019) 1818(12):2246–57. doi: 10.1158/1535-7163.MCT-18-1146 31395689

[75] Zhao G Wei R Feng L Wu Y He F Xiao M. Lenalidomide enhances the efficacy of anti-BCMA CAR-T treatment in relapsed/refractory multiple myeloma: A case report and revies of the literature. Cancer Immunol Immunother (2022) 71(1):39–44. doi: 10.1007/s00262-021-02959-8 34003300 PMC8738460

[76] Desantis V Savino FD Scaringella A Potenza MA Nacci C Frassanito MA. The leading role of the immune microenvironment in multiple myeloma: A new target with a great prognostic and clinical value. J Clin Med (2022) 11(9):2513. doi: 10.3390/jcm11092513 35566637 PMC9105926

[77] Holthof LC Mutis T. Challenges for immunotherapy in multiple myeloma: Bone marrow microenvironment-mediated immune suppression and immune resistance. Cancers (Basel) (2020) 12(4):988. doi: 10.3390/cancers12040988 32316450 PMC7226482

[78] Dhakal B Hari PN Usmani SZ Hamadani M. Chimeric antigen receptor T cell therapy in multiple myeloma: Promise and challenges. Bone Marrow Transplant (2021) 56(1):9–19. doi: 10.1038/s41409-020-01023-w 32770147

[79] An G Acharya C Feng X Wen K Zhong M Zhang L. Osteoclasts promote immune suppressive microenvironment in multiple myeloma: Therapeutic implication. Blood (2016) 128(12):1590–603. doi: 10.1182/blood-2016-03-707547 PMC5034739 27418644

[80] Görgün GT Whitehill G Anderson JL Hideshima T Maguire C Laubach J. Tumor-promoting immune-suppressive myeloid-derived suppressor cells in the multiple myeloma microenvironment in humans. Blood (2013) 121(15):2975–87. doi: 10.1182/blood-2012-08-448548 PMC3624943 23321256

[81] Sun J Park C Guenthner N Gurley S Zhang L Lubben B. Tumor-associated macrophages in multiple myeloma: Advances in biology and therapy. J Immunother Cancer (2022) 10(4):e003975. doi: 10.1136/jitc-2021-003975 35428704 PMC9014078

[82] André T Najar M Stamatopoulos B Pieters K Pradier O Bron D. Immune impairments in multiple myeloma bone marrow mesenchymal stromal cells. Cancer Immunol Immunother (2015) 64(2):213–24. doi: 10.1007/s00262-014-1623-y PMC11029797 25341809

[83] Romano A Parrinello NL Simeon V Puglisi F La Cava P Bellofiore C. High-density neutrophils in MGUS and multiple myeloma are dysfunctional and immune-suppressive due to increased STAT3 downstream signaling. Sci Rep (2020) 10(1):1983. doi: 10.1038/s41598-020-58859-x 32029833 PMC7005058

[84] Brimnes MK Svane IM Johnsen HE. Impaired functionality and phenotypic profile of dendritic cells from patients with multiple myeloma. Clin Exp Immunol (2006) 144(1):76–84. doi: 10.1111/j.1365-2249.2006.03037.x 16542368 PMC1809645

[85] Pilcher W Thomas BE Bhasin SS Jayasinghe RG Yao L Gonzalez-Kozlova E. Cross center single-cell RNA sequencing study of the immune microenvironment in rapid progressing multiple myeloma. NPJ Genom Med (2023) 8(1):3. doi: 10.1038/s41525-022-00340-x 36702834 PMC9879959

[86] Yan H He D Qu J Liu Y Xu R Gu H. Interleukin-32γ promotes macrophage-mediated chemoresistance by inducing CSF1-dependent M2 macrophage polarization in multiple myeloma. Cancer Immunol Immunother (2023) 72(2):327–38. doi: 10.1007/s00262-022-03241-1 PMC10991222 35881196

[87] Sun M Qiu S Xiao Q Wang T Tian X Chen C. Synergistic effects of multiple myeloma cells and tumor-associated macrophages on vascular endothelial cells. vitro Med Oncol (2020) 37(11):99. doi: 10.1007/s12032-020-01426-1 33040185

[88] Wang Z Zhang L Wang H Xiong S Li Y Tao Q. Tumor-induced CD14+HLA-DR (-/low) myeloid-derived suppressor cells correlate with tumor progression and outcome of therapy in multiple myeloma patients. Cancer Immunol Immunother (2015) 64(3):389–99. doi: 10.1007/s00262-014-1646-4 PMC11028624 25548095

[89] Giallongo C Tibullo D Parrinello NL La Cava P Di Rosa M Bramanti V. Granulocyte-like myeloid derived suppressor cells (G-MDSC) are increased in multiple myeloma and are driven by dysfunctional mesenchymal stem cells (MSC). Oncotarget (2016) 7(52):85764–75. doi: 10.18632/oncotarget.7969 PMC5349872 26967390

[90] Petersson J Askman S Pettersson A Wichert S Hellmark T Johansson ACM. Bone marrow neutrophils of multiple myeloma patients exhibit myeloid-derived suppressor cell activity. J Immunol Res (2021) 2021:6344344. doi: 10.1155/2021/6344344 34414242 PMC8369183

[91] Li M Lin C Deng H Strnad J Bernabei L Vogl DT. A novel peptidylarginine deiminase 4 (PAD4) inhibitor BMS-P5 blocks formation of neutrophil extracellular traps and delays progression of multiple myeloma. Mol Cancer Ther (2020) 19(7):1530–8. doi: 10.1158/1535-7163.MCT-19-1020 PMC7335350 32371579

[92] de Jong MME Kellermayer Z Papazian N Tahri S Hofste Op Bruinink D Hoogenboezem R. The multiple myeloma microenvironment is defined by an inflammatory stromal cell landscape. Nat Immunol (2021) 22(6):769–80. doi: 10.1038/s41590-021-00931-3 34017122

[93] Swamydas M Murphy EV Ignatz-Hoover JJ Malek E Driscoll JJ. Deciphering mechanisms of immune escape to inform immunotherapeutic strategies in multiple myeloma. J Hematol Oncol (2022) 15(1):17. doi: 10.1186/s13045-022-01234-2 35172851 PMC8848665

[94] Benson DM Jr Bakan CE Mishra A Hofmeister CC Efebera Y Becknell B. The PD-1/PD-L1 axis modulates the natural killer cell versus multiple myeloma effect: a therapeutic target for CT-011, a novel monoclonal anti-PD-1 antibody. Blood (2010) 116(13):2286–94. doi: 10.1182/blood-2010-02-271874 PMC3490105 20460501

[95] Li X Chen M Wan Y Zhong L Han X Chen X. Single-cell transcriptome profiling reveals the key role of ZNF683 in natural killer cell exhaustion in multiple myeloma. Clin Transl Med (2022) 12(10):e1065. doi: 10.1002/ctm2.1065 36245253 PMC9574488

[96] Pazina T MacFarlane AW4 Bernabei L Dulaimi E Kotcher R Yam C. Alterations of NK cell phenotype in the disease course of multiple myeloma. Cancers (Basel) (2021) 13(2):226. doi: 10.3390/cancers13020226 33435153 PMC7827733

[97] Chan AC Neeson P Leeansyah E Tainton K Quach H Prince HM. Natural killer T cell defects in multiple myeloma and the impact of lenalidomide therapy. Clin Exp Immunol (2014) 175(1):49–58. doi: 10.1111/cei.12196 24032527 PMC3898554

[98] Moon EK Wang LC Dolfi DV Wilson CB Ranganathan R Sun J. Multifactorial T-cell hypofunction that is reversible can limit the efficacy of chimeric antigen receptor-transduced human T cells in solid tumors. Clin Cancer Res (2014) 20(16):4262–73. doi: 10.1158/1078-0432.CCR-13-2627 PMC4134701 24919573

[99] Holthof LC van der Schans JJ Katsarou A Poels R Gelderloos AT Drent E. Bone marrow mesenchymal stromal cells can render multiple myeloma cells resistant to cytotoxic machinery of CAR T cells through inhibition of apoptosis. Clin Cancer Res (2021) 27(13):3793–803. doi: 10.1158/1078-0432.CCR-20-2188 33883175

[100] An N Hou YN Zhang QX Li T Zhang QL Fang C. Anti-multiple myeloma activity of nanobody-based anti-CD38 chimeric antigen receptor T cells. Mol Pharm (2018) 15(10):4577–88. doi: 10.1021/acs.molpharmaceut.8b00584 30185037

[101] Cook J Acosta-Medina AA Peng KW Lacy M Russell S. Oncolytic virotherapy - forging its place in the immunomodulatory paradigm for multiple myeloma. Cancer Treat Res Commun (2021) 29:100473. doi: 10.1016/j.ctarc.2021.100473 34673439

[102] Hu Y Wang J Wei G Yu J Luo Y Shi J. A retrospective comparison of allogenic and autologous chimeric antigen receptor T cell therapy targeting CD19 in patients with relapsed/refractory acute lymphoblastic leukemia. Bone Marrow Transplant (2019) 54(8):1208–17. doi: 10.1038/s41409-018-0403-2 30518980

[103] Mailankody S Matous JV Chhabra S Liedtke M Sidana S Oluwole OO. Allogeneic BCMA-targeting CAR T cells in relapsed/refractory multiple myeloma: Phase 1 UNIVERSAL trial interim results. Nat Med (2023). doi: 10.1038/s41591-022-02182-7 36690811

[104] Lança T Correia DV Moita CF Raquel H Neves-Costa A Ferreira C. The MHC class ib protein ULBP1 is a nonredundant determinant of leukemia/lymphoma susceptibility to gammadelta T-cell cytotoxicity. Blood (2010) 115(12):2407–11. doi: 10.1182/blood-2009-08-237123 20101024

[105] Pistoia V Tumino N Vacca P Veneziani I Moretta A Locatelli F. Human γδ T-cells: From surface receptors to the therapy of high-risk leukemias. Front Immunol (2018) 9:984. doi: 10.3389/fimmu.2018.00984 29867961 PMC5949323

[106] Rozenbaum M Meir A Aharony Y Itzhaki O Schachter J Bank I. Gamma-delta CAR-T cells show CAR-directed and independent activity against leukemia. Front Immunol (2020) 11:1347. doi: 10.3389/fimmu.2020.01347 32714329 PMC7343910

[107] Airoldi I Bertaina A Prigione I Zorzoli A Pagliara D Cocco C. γδ T-cell reconstitution after HLA-haploidentical hematopoietic transplantation depleted of TCR-αβ+/CD19+ lymphocytes. Blood (2015) 125(15):2349–58. doi: 10.1182/blood-2014-09-599423 PMC4440890 25612623

[108] Nishimoto KP Barca T Azameera A Makkouk A Romero JM Bai L. Allogeneic CD20-targeted γδ T cells exhibit innate and adaptive antitumor activities in preclinical b-cell lymphoma models. Clin Transl Immunol (2022) 11(2):e1373. doi: 10.1002/cti2.1373 PMC8809437 35136603

[109] Handgretinger R Schilbach K. The potential role of γδ T cells after allogeneic HCT for leukemia. Blood (2018) 131(10):1063–72. doi: 10.1182/blood-2017-08-752162 29358176

[110] Chu J Deng Y Benson DM He S Hughes T Zhang J. CS1-specific chimeric antigen receptor (CAR)-engineered natural killer cells enhance *in vitro* and *in vivo* antitumor activity against human multiple myeloma. Leukemia (2014) 28(4):917–27. doi: 10.1038/leu.2013.279 PMC3967004 24067492

[111] Leivas A Valeri A Córdoba L García-Ortiz A Ortiz A Sánchez-Vega L. NKG2D-CAR-transduced natural killer cells efficiently target multiple myeloma. Blood Cancer J (2021) 11(8):146. doi: 10.1038/s41408-021-00537-w 34392311 PMC8364555

[112] Luanpitpong S Poohadsuan J Klaihmon P Issaragrisil S. Selective cytotoxicity of single and dual anti-CD19 and anti-CD138 chimeric antigen receptor-natural killer cells against hematologic malignancies. J Immunol Res (2021) 2021:5562630. doi: 10.1155/2021/5562630 34337077 PMC8289607

[113] Munshi NC Anderson LD Jr Shah N Madduri D Berdeja J Lonial S. Idecabtagene vicleucel in relapsed and refractory multiple myeloma. N Engl J Med (2021) 384(8):705–16. doi: 10.1056/NEJMoa2024850 33626253

[114] Huang R Wang X Zhang X. Unity brings strength: Combination of CAR-T cell therapy and HSCT. Cancer Lett (2022) 549:215721. doi: 10.1016/j.canlet.2022.215721 35537573

[115] Wei J Xiao M Mao Z Wang N Cao Y Xiao Y. Outcome of aggressive b-cell lymphoma with TP53 alterations administered with CAR T-cell cocktail alone or in combination with ASCT. Signal Transduct Target Ther (2022) 7(1):101. doi: 10.1038/s41392-022-00924-0 35399106 PMC8995369

[116] Wang T Xu L Gao L Tang G Chen L Chen J. Chimeric antigen receptor T-cell therapy combined with autologous stem cell transplantation improved progression-free survival of relapsed or refractory diffuse Large b-cell lymphoma patients: A single-center, retrospective, cohort study. Hematol Oncol (2022) . 40(4):637–44. doi: 10.1002/hon.2975 35141937

[117] Wang T Gao L Wang Y Zhu W Xu L Wang Y. Hematopoietic stem cell transplantation and chimeric antigen receptor T cell for relapsed or refractory diffuse large b-cell lymphoma. Immunotherapy (2020) 12(13):997–1006. doi: 10.2217/imt-2020-0075 32752910 PMC7546158

[118] Ye M Gao L Wang T Yu J Gui J Yang J. CD19 chimeric antigen receptor T-cell therapy following autologous stem cell transplantation against relapsed or refractory burkitt lymphoma/leukemia: A case report and literature review. Front Oncol (2022) 12:932254. doi: 10.3389/fonc.2022.932254 36353549 PMC9639856

[119] Pocaterra A Catucci M Mondino A. Adoptive T cell therapy of solid tumors: time to team up with immunogenic chemo/radiotherapy. Curr Opin Immunol (2021) 74:53–9. doi: 10.1016/j.coi.2021.10.004 34743069

[120] Zhang X Wu J Qiao L Chen L Chen C Zhang H. Case report: Cryoablation as a novel bridging strategy prior to CAR-T cell therapy for b cell malignancies with bulky disease. Front Oncol (2023) 13:1008828. doi: 10.3389/fonc.2023.1008828 36776338 PMC9911860

[121] Spiotto M Fu YX Weichselbaum RR. The intersection of radiotherapy and immunotherapy: mechanisms and clinical implications. Sci Immunol (2016) 1(3):EAAG1266. doi: 10.1126/sciimmunol.aag1266 28018989 PMC5171206

[122] Sun T Li Y Yang Y Liu B Cao Y Yang W. Enhanced radiation-induced immunogenic cell death activates chimeric antigen receptor T cells by targeting CD39 against glioblastoma. Cell Death Dis (2022) 13(10):875. doi: 10.1038/s41419-022-05319-1 36245000 PMC9573869

[123] Yang J He J Zhang X Li J Wang Z Zhang Y. Next-day manufacture of a novel anti-CD19 CAR-T therapy for b-cell acute lymphoblastic leukemia: first-in-human clinical study. Blood Cancer J (2022) 12(7):104. doi: 10.1038/s41408-022-00694-6 35798714 PMC9262977

[124] Ghassemi S Nunez-Cruz S O’Connor RS Fraietta JA Patel PR Scholler J. Reducing ex vivo culture improves the antileukemic activity of chimeric antigen receptor (CAR) T cells. Cancer Immunol Res (2018) 6(9):1100–9. doi: 10.1158/2326-6066.CIR-17-040 PMC8274631 30030295

[125] Costa LJ Kumar S Atrash S Liedtke M Kaur G Derman B. Results from the First Phase 1 Clinical Study of the B-Cell Maturation Antigen (BCMA) Nex T Chimeric Antigen Receptor (CAR) T Cell Therapy CC-98633/BMS-986354 in Patients (pts) with Relapsed/Refractory Multiple Myeloma (RRMM). Blood (2022) 140(Supplement 1):1360–2. doi: 10.1182/blood-2022-160038

[126] Magnani CF Gaipa G Lussana F Belotti D Gritti G Napolitano S. Sleeping beauty-engineered CAR T cells achieve antileukemic activity without severe toxicities. J Clin Invest (2020) 130(11):6021–33. doi: 10.1172/JCI138473 PMC7598053 32780725

[127] Suematsu M Yagyu S Nagao N Kubota S Shimizu Y Tanaka M. PiggyBac transposon-mediated CD19 chimeric antigen receptor-T cells derived from CD45RA-positive peripheral blood mononuclear cells possess potent and sustained antileukemic function. Front Immunol (2022) 13:770132. doi: 10.3389/fimmu.2022.770132 35154098 PMC8829551

[128] Lock D Monjezi R Brandes C Bates S Lennartz S Teppert K. Automated, scaled, transposon-based production of CAR T cells. J Immunother Cancer (2022) 10(9):e005189. doi: 10.1136/jitc-2022-005189 36096530 PMC9472140

[129] Prommersberger S Reiser M Beckmann J Danhof S Amberger M Quade-Lyssy P. CARAMBA: A first-in-human clinical trial with SLAMF7 CAR-T cells prepared by virus-free sleeping beauty gene transfer to treat multiple myeloma. Gene Ther (2021) 28(9):560–71. doi: 10.1038/s41434-021-00254-w PMC8455317 33846552

[130] Magnani CF Mezzanotte C Cappuzzello C Bardini M Tettamanti S Fazio G. Preclinical efficacy and safety of CD19CAR cytokine-induced killer cells transfected with sleeping beauty transposon for the treatment of acute lymphoblastic leukemia. Hum Gene Ther (2018) 29(5):602–13. doi: 10.1089/hum.2017.207 29641322

[131] Lu Y Xue J Deng T Zhou X Yu K Deng L. Safety and feasibility of CRISPR-edited T cells in patients with refractory non-small-cell lung cancer. Nat Med (2020) 26(5):732–40. doi: 10.1038/s41591-020-0840-5 32341578

[132] Chari A Vogl DT Jagannath S Jasielec J Unger TJ DeCastro A. Selinexor-based regimens for the treatment of myeloma refractory to chimeric antigen receptor T cell therapy. Br J Haematol (2020) 189(4):e126–30. doi: 10.1111/bjh.16550 32124443

[133] Parrondo RD Sam K Rasheed A Alegria V Sher T Roy V. Subsequent anti-myeloma therapy after idecabtagene vicleucel treatment in patients with relapsed/refractory multiple myeloma: A single center analysis. Blood Cancer J (2022) 12(4):66. doi: 10.1038/s41408-022-00662-0 35440072 PMC9019050

[134] Van Oekelen O Nath K Mouhieddine TH Farzana T Aleman A Melnekoff DT. Interventions and outcomes of multiple myeloma patients receiving salvage treatment after BCMA-directed CAR T therapy. Blood (2022). doi: 10.1182/blood.2022017848 PMC10082354 36327160

[135] Zhao WH Wang BY Chen LJ Fu WJ Xu J Liu J. Four-year follow-up of LCAR-B38M in relapsed or refractory multiple myeloma: A phase 1, single-arm, open-label, multicenter study in China (LEGEND-2). J Hematol Oncol (2022) 15(1):86. doi: 10.1186/s13045-022-01301-8 35794616 PMC9261106

[136] Chen D Wang X Chen Z Jiang S Jiang H Fu W. Subsequent anti-myeloma therapy after maturation antigen (BCMA) chimeric antigen receptor (CAR)-T cell (HDS269B) treatment in patients with relapsed/refractory multiple myeloma. Am J Hematol (2022) 97(12):E478–81. doi: 10.1002/ajh.26745 36197023

[137] Trudel S Cohen AD Krishnan AY Fonseca R Spencer A Berdeja JG. Cevostamab monotherapy continues to show clinically meaningful activity and manageable safety in patients with heavily pre-treated relapsed/refractory multiple myeloma (RRMM): Updated results from an ongoing phase I study. Blood (2021) 138(Supplement 1):157. doi: 10.1182/blood-2021-147983

[138] Kang C. Teclistamab: First approval. Drugs (2022) 82(16):1613–9. doi: 10.1007/s40265-022-01793-1 PMC9646474 36352205

[139] Chari A Touzeau C Schinke C Minnema MC Berdeja J Oriol A. Talquetamab, a g protein-coupled receptor family c group 5 member d x CD3 bispecific antibody, in patients with relapsed/refractory multiple myeloma (RRMM): Phase 1/2 results from MonumenTAL-1. Blood (2022) 140(Supplement 1):384–7. doi: 10.1182/blood-2022-159707

[140] Longcor J Callander N Oliver K Chanan-Khan A Ailawadhi S. Iopofosine I-131 treatment in late-line patients with relapsed/refractory multiple myeloma post anti-BCMA immunotherapy. Blood Cancer J (2022) 12(9):130. doi: 10.1038/s41408-022-00725-2 36068232 PMC9448804

